# Cross-section measurements for ^68^Zn(*p*,2*p*)^67^Cu and ^68^Zn(*p*,2*n*)^67^Ga reactions using a newly developed separation method for the superposed $\gamma$-ray spectra

**DOI:** 10.1038/s41598-023-38483-1

**Published:** 2023-07-13

**Authors:** Myung-Hwan Jung, Won-Je Cho, Hye Min Jang, Kwon-Soo Chun, Jae Sang Lee, Yong Seok Hwang, Sang Wook Kim, Jun Kue Park

**Affiliations:** 1 Korea Multi-purpose Accelerator Complex, Korea Atomic Energy Research Institute, Gyeongju, 38180 Korea; 2 Department of Advanced Materials Chemistry, Dongguk University, Gyeongju, 38066 Republic of Korea

**Keywords:** Experimental nuclear physics, Scientific data, Characterization and analytical techniques

## Abstract

We have developed a new analytical peak separation analysis for superposed $\gamma$-ray peaks on $^{67}$Cu and $^{67}$Ga to measure the $^{68}$Zn(*p*,2*p*)$^{67}$Cu and $^{68}$Zn(*p*,2*n*)$^{67}$Ga reactions, unlike in most previous works that were employing a radiochemical separation to measure them. Based on the nuclear data such as the $\gamma$-ray intensity and the half-life for each nuclide, we may develop a new analytical method that enables us to estimate the respective counts arising from each nuclide, thereby obtaining the nuclear reactions. The newly developed analytical method can universally be applied to separate the superposed $\gamma$-ray spectra of any two nuclides, especially superior in separating the nuclides with different half-lives. In comparison with the data in the literature, the two reactions in the present work are in good agreement with those of some previous works. In addition, we compared the present $^{68}$Zn(*p*,2*n*)$^{67}$Ga reaction without the peak separation to the data in the literature without the chemical separation, and find that a good agreement is evident, enhancing the reliability of the $^{68}$Zn(*p*,*x*)$^{65}$Zn and $^{68}$Zn(*p*,3*n*)$^{66}$Ga reactions, which are further measured in the present work

## Introduction

Copper-67 has a half-life $t_{1/2}$ = 61.83 h and emits $\beta ^{-}$ radiation with a mean energy of 141 keV giving a higher linear energy transfer than that of $\gamma$-rays with energies of 93.3 keV and 184.6 keV when it decays to $^{67}$Zn^1–6^. Among some nuclear reactions for $^{67}$Cu, $^{70}$Zn(*p*,$\alpha$)$^{67}$Cu reaction may exhibit the maximum value of 14.9 mb at 14.8 MeV^7^. However, there is some difficulty with application due to the low natural abundance (0.61%) of the $^{70}$Zn nuclide. On the other hand, the cross-section of $^{67}$Cu via the nuclear reaction of $^{68}$Zn(*p*,2*p*) exhibits the highest yield in the range from 40 MeV to 100 MeV compared to other isotopes (see Table 1)^1–6,8,10,11^. Excitation functions of (*p*,*xp*) reactions where *x* is an integer may undergo the complexity of the problem with increasing *x* so that one needs to figure out the simpler reactions to understand such the complex reactions^1,2^.

**Table 1:** Summary of possible representative nuclear reaction routes for $^{67}$Cu production.

Target materials	Isotopic abundance (%)	Projectile ions	Production route	Energy range (MeV)	$\sigma _{max}$ [mb] (at an energy [MeV])	References
^nat^Zn	–	*p*	(*p*,*x*)	47.5-99.2	4.1 (67.0)	^6^
		*d*	(*d*,2*p*)	10.0–50.0	4.3 (45.4)	^9^
$^{68}$Zn	18.45	*p*	(*p*,2*p*)	20.0–85.0	11.4 (70.2)	^5^
		*d*	(*d*,$^{3}$He)	11.0–15.4	0.57 (15.4)	^10^
		*p*	(*p*,$\alpha$)	7.5–35.0	14.9 (14.8)	^8^
$^{70}$Zn	0.61	*p*	(*p*,*x*)	44.7-67.8	21.5 (67.8)	^7^
		*d*	(*d*,$\alpha$ $n$)	10.1–19.7	27 (18.9)	^11^

Representative radioactive isotopes of copper may include $^{64}$Cu and $^{67}$Cu. These are used as theranostic pair radionuclides owing to their complementary characteristics^12^. $^{64}$Cu is a positron-emitting nuclide having a relatively long half-life of 2.7 h^13^. Thus, it can be used for long-term positron emission tomography imaging and can determine the dose of a tracer labeled with $^{67}$Cu.

In most previous works, they attempted to obtain the cross sections of $^{68}$Zn(*p*, 2*p*) via a radiochemical separation method that can make a loss in separation efficiency^1–5,8,14^. In addition, the uncertainties may increase during the radiochemical procedure for separating the activated radionuclide of interest from the matrix activity^15^. In assessing the $^{64}$Cu and $^{67}$Cu contents, the high potential $^{ 67}$Ga contamination may give us the data being faithless^14^. So far, most previous works made a radiochemical separation not avoiding the issues mentioned above or did not make the separation only obtaining the $^{68}$Zn(*p*,2*n*)$^{67}$Ga reactions. Hence, to exactly assess the $^{64}$Cu and $^{67}$Cu contents, the $\gamma$-ray spectrum for $^{67}$Cu which is superposed with that for $^{67}$Ga should be analyzed.

For the analytical separation previously reported^6,14^, the equations were derived from nuclear data of the $\gamma$-ray intensity distinct for each nuclide. However, this approach did not consider slightly different half-lives of the two nuclides with $^{67}$Cu and $^{67}$Ga (see Table 2)^16^. Upon increasing waiting time after irradiation, the $t_{1/2}$ data results in the $\gamma$-counts being wrongly separated. In other words, the cross sections of $^{67}$Cu and $^{67}$Ga may be changed, depending on when the spectra are measured. Thus, a more concrete separation analysis should be constructed so that the nuclear data of not only the $\gamma$-ray intensity but also the half-life for each nuclide are considered. Here, we for the first time have derived the separation analysis fully considering the two nuclear data. Furthermore, we systematically investigated how much the data can be deviated depending on two different separation approaches and none of the separation for $^{67}$Ga, comparing those with the data in the literature. The present work also reports the $^{68}$Zn(*p*,*x*)$^{65}$Zn and $^{68}$Zn(*p*,3*n*)$^{66}$Ga reactions.

**Table 2:** Decay data to radionuclides of interest, data extracted from the NuDat 3.0 database from National Nuclear Data Center^16^.

Radionuclide	Half-life	E$_{\gamma }$ (keV)	I$_{\gamma }$ [%]
$^{67}$Cu	61. 83 h (12)	91.266 (5)	7.00 (10)
		93.311 (5)	16.10 (20)
		184.577 (10)	48.7 (3)
		208.951 (10)	0.115 (5)
		300.219 (10)	0.797 (11)
		393.529 (10)	0.220 (8)
$^{67}$Ga	72.281 h (12)	91.266 (5)	3.11 (4)
		93.310 (5)	38.81 (3)
		184.576 (10)	21.410 (10)
		208.950 (10)	2.460 (10)
		300.217 (10)	16.64 (12)
		393.527 (10)	4.56 (24)
$^{65}$Zn	243.93 d (9)	511.0	2.842 (14)
		115.539 (2)	50.04 (10)
$^{66}$Ga	9.43 h (3)	511.0	114 (8)
		833.5324 (21)	5.9 (3)
		1039.220 (3)	37.0 (20)
$^{24}$Na (monitor reaction)	14.997 h (12)	1368.626 (5)	99.9936 (15)
		2754.07 (11)	99.855 (5)

## Peak separation analysis

We previously developed a peak separation method based on distinct $\gamma$-ray constants for each peak of $^{67}$Cu and $^{67}$Ga, but not considering distinct decay constants of the nuclides^6^. A cooling time $t_{w}$ is indispensable during acquiring the data, so it causes a discrepancy in the separated $\gamma$-ray counting upon longer $t_{w}$, although $^{67}$Cu and $^{67}$Ga have close-lying half-lives for $^{67}$Cu ($T_{1/2}$ = 61.9 h) and $^{67}$Ga ($T_{1/2}$ = 78.3 h). Moreover, imagine the case when two half-lives are greatly different by as much as one order of magnitude. Then, the counting from each nuclide exhibits a greater difference upon increasing $t_{w}$, although the $\gamma$-ray intensities of some peaks from a nuclide are independent of the time. Hence, in this work, we develop a more concrete separation method further considering the decay constants for the first time as far as we know.

### Peak separation analysis with $\gamma$-ray intensity

First, we recall a separation process reflecting only the distinct $\gamma$-ray intensities of two nuclides of $^{67}$Cu and $^{67}$Ga, which were previously developed by our work^6^. This separation process is started by defining that the ratio of the $\gamma$-ray intensities is the same as that of corrected counts for the nuclides. Second, a superposed $\gamma$-ray peak consists of two kinds of counting from two nuclides of $^{67}$Cu and $^{67}$Ga. Using these conditions, we may write each $\gamma$-ray counting^6^,

1 $$\begin{aligned} A_{1}= & {} \frac{I_{A\gamma 1}(I_{B\gamma 1}\cdot C_{2,tot}-I_{B\gamma 2} \cdot C_{1,tot})}{I_{A\gamma 2}\cdot I_{B\gamma 1}-I_{A\gamma 1}\cdot I_{B\gamma 2}}, \end{aligned}$$

2 $$\begin{aligned} B_{1}= & {} \frac{I_{B\gamma 1}(I_{A\gamma 2}\cdot C_{1,tot}-I_{A\gamma 1} \cdot C_{2,tot})}{I_{A\gamma 2}\cdot I_{B\gamma 1}-I_{A\gamma 1} \cdot I_{B\gamma 2}}, \end{aligned}$$

where the counts for each nuclide of $A_{1}$ and $B_{1}$ do not depend on time with variables of the time-independent total counts of $C_{1,tot}$ and $C_{2,tot}$. We denote this separation analysis as Method I.

### Peak separation analysis with both $\gamma$-ray intensity and decay constant

We now introduce a new separation analysis which is further considered with different decay constants of $\lambda _{A}$ and $\lambda _{B}$ from nuclides *A* and *B*, respectively. Let us consider a total count for a superposed $\gamma$-ray peak, i.e., a peak with $E_{\gamma }$ = 184.6 keV as being denoted by $C_{1,tot}$(*t*), which consists of two kinds of counting from two nuclides of $^{67}$Cu and $^{67}$Ga as being $A_{1}$(*t*) and $B_{1}$(*t*), respectively. We may expand this relation to other $\gamma$-ray peaks, and thus given by,

3 $$\begin{aligned} C_{i,tot}(t)=A_{i}(t)+B_{i}(t), \qquad i=1,2,\ldots ,n, \end{aligned}$$

We may explicitly write a count for a $\gamma$-ray peak with an energy of *i*, $A_{i}$(*t*), for a nuclide,

4 $$\begin{aligned} A_{i}(t)=\frac{N_{0}\sigma _{A}\Phi }{\lambda _{A}} I_{A,\gamma i}(1-e^{-\lambda _{A}t_{i}}) \cdot e^{-\lambda _{A}t_{w}}(1-e^{-\lambda _{A}t_{m}}), \qquad i=1,2,\ldots ,n, \end{aligned}$$

where $N_{0}$ denotes the initial number of nuclei , $\sigma _{A}$ the reaction cross section of the nuclide *A*, $\Phi$ the flux of the incident particles, $\lambda _{A}$ decay constant of the half-life ($t_{1/2}$) of the radioactive decay from the nuclide *A*, $I_{A,\gamma i}$ the intensity of the $\gamma$-ray for a peak *i* of the nuclide *A*, $t_{i}$ the irradiation or activation time, $t_{w}$ a waiting time or cooling time, and $t_{m}$ the measurement time. Similarly, for the nuclide $^{67}$Ga, we may obtain $B_{i}$(t) by replacing $\lambda _{A}$ to $\lambda _{B}$ and $I_{A,\gamma i}$ to $I_{B,\gamma i}$.

For the timing factor, the irradiation time $t_{i}$ and the measurement time $t_{m}$ can be negligible due to its relatively short time compared to the waiting time $t_{w}$ ($t_{i}$, $t_{m}$ $\ll$ $t_{w}$), thus assuming that $t_{i}$ and $t_{m}$ do not affect the peak separation process. Hence, we may simply consider the variable $t_{w}$ in the separation process.

5 $$\begin{aligned} C_{i,tot}(t_{w1})= & {} A_{i}(t_{w1})+B_{i}(t_{w1}), \qquad i=1,2,\ldots ,n, \end{aligned}$$

6 $$\begin{aligned} C_{i,tot}(t_{w2})= & {} A_{i}(t_{w2})+B_{i}(t_{w2}) \nonumber \\= & {} A_{i}(t_{w1}) e^{-\lambda _{A}(t_{w2}-t_{w1})} +B_{i}(t_{w1}) e^{-\lambda _{B}(t_{w2}-t_{w1})}, \qquad i=1,2,\ldots ,n, \end{aligned}$$

7 $$\begin{aligned} \frac{A_{i}(t_{w2})}{A_{1}(t_{w1})}= & {} \frac{I_{A\gamma i}}{I_{A\gamma 1}} e^{-\lambda _{A}(t_{w2}-t_{w1})}, \quad \frac{B_{i}(t_{w2})}{B_{1}(t_{w1})}=\frac{I_{B\gamma i}}{I_{B\gamma 1}} e^{-\lambda _{B}(t_{w2}-t_{w1})} \end{aligned}$$

Comparing the ratio of $C_{2,\mathrm tot}$($t_{w2}$)/$C_{1,\mathrm tot}$($t_{w1}$) at two different waiting times, i.e., $t_{w1}$ and $t_{w2}$, we may obtain useful relations of Eq. (7) by using Eqs. (4) and (6). Plugging Eqs. (7) into (6) and then solving simultaneously Eqs. (5) and (6), we may obtain the following equations for two peaks with *i* = 1 and 2,

8 $$\begin{aligned} A_{1}(t_{w1})= & {} \frac{e^{\lambda _{A}t_{w2}}I_{A\gamma 1}\big \{e^{\lambda _{B}t_{w2}}C_{2,tot}(t_{w2})I_{B\gamma 1}-e^{\lambda _{B}t_{w1}}C_{1,tot}(t_{w1})I_{B\gamma 2} \}}{e^{\lambda _{A}t_{w1}+\lambda _{B}t_{w2}}I_{A\gamma 2}I_{B\gamma 1}-e^{\lambda _{A}t_{w2}+\lambda _{B}t_{w1}}I_{A\gamma 1}I_{B\gamma 2}}, \end{aligned}$$

9 $$\begin{aligned} B_{1}(t_{w1})= & {} \frac{e^{\lambda _{B}t_{w2}}I_{B\gamma 1}\big \{e^{\lambda _{A}t_{w2}}C_{2,tot}(t_{w2})I_{A\gamma 1}-e^{\lambda _{A}t_{w1}}C_{1,tot}(t_{w1})I_{A\gamma 2} \}}{e^{\lambda _{A}t_{w2}+\lambda _{B}t_{w1}}I_{A\gamma 1}I_{B\gamma 2}-e^{\lambda _{A}t_{w1}+\lambda _{B}t_{w2}}I_{A\gamma 2}I_{B\gamma 1}}. \end{aligned}$$

We denote this separation analysis as Method II. From Eqs. (8) and (9), we may obtain each separated $\gamma$-ray counting, and thus obtain cross sections for $^{67}$Cu and $^{67}$Ga. We may ensure that Eqs. (8) and (9) correspond to Eqs. (1) and (2), respectively, when the decay constants do not depend on the waiting time $t_{w}$, i.e., $t_{w1}$ = $t_{w2}$. It should be noted that $C_{i,tot}$(t) is a corrected total counting, i.e., $C_{i,tot}$(t) = $S_{i}$(t)/$\varepsilon _{\gamma ,i}$, where $S_{i}$(t) is the number of counts recorded by the detection system at a time and $\epsilon _{\gamma ,i}$ is the $\gamma$-counting efficiency of the detector. In the next section, we will exhibit the $^{68}$Zn(*p*,2*p*) reaction by applying two distinct analyses of Method I and Method II.

## Experimental results and discussion

### $^{68}$Zn(*p*,2*p*)$^{67}$Cu reaction

**Figure 1:**
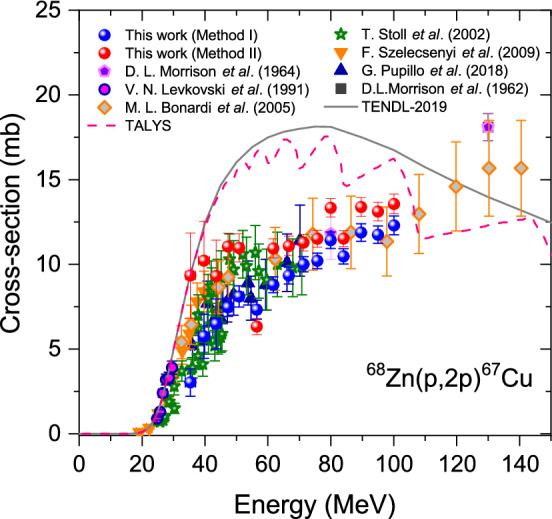
Measured excitation functions for the $\gamma$-ray emitted following the $^{68}$Zn(*p*,2*p*)$^{67}$Cu reaction as a function of incident proton energy. We plot the data using the developed separation processes for the superposed $\gamma$-ray as denoted by Method I and Method II. We also include theoretical calculations using a statistical model codes as denoted by TENDL-2019 and TALYS^27^ as well as the data from literature.

We display the proton-induced excitation function of $^{68}$Zn(*p*,2*p*)$^{67}$Cu based on the analytical separation processes that have been developed, together with previous literature data and the TENDL-2019 library, as shown in Fig. 1. We compare the data obtained by our two analytical separation processes (Table 3), in which the data obtained by Method II are somewhat greater than that obtained by Method I. The present $^{68}$Zn(*p*,2*p*)$^{67}$Cu reactions with Method I and Method II are in good agreement with those reported by Pupillo et al.^5^ and Stoll et al.^4^, respectively, within their quoted uncertainties. Taking into account that Method II reflects all parameters we need to consider, we may expect that the data from Method II would give more accurate values. Most previous works^3–5,8^ provide a detailed systematic study on the reaction up to $\sim$70 MeV, above which, however, none of the detailed data was reported. So far, all works provide the reaction by employing a radiochemical process^1–5,8^. We for the first time provide detailed data up to 100 MeV with the developed separation processes that feature no loss in separation efficiency, unlike in a radiochemical process.

To estimate the beam flux, the monitor reaction we employed was ^nat^Al(*p*,*x*)$^{24}$Na, the data recommended by IAEA^17^. Stoll et al.^4^ estimated the beam current via the ^nat^Cu(*p*,*xn*)$^{62,63}$Zn and the $^{27}$Al(*p*, *x*)$^{22}$Na reactions. On the other hand, Pupillo et al. employed the monitor reactions of ^nat^Ni(*p*,*x*)$^{57}$Ni and ^nat^Al(*p*,*x*)$^{22}$Na^5^. Compared with previous and present monitor reactions, the Al reaction was used in common for measuring the beam flux.

**Table 3:** Measured cross-sections of the radionuclides of interest in the $^{68}$Zn(*p*,*x*) production route.

Energy	$^{68}$Zn(*p*,2*p*)$^{67}$Cu	$^{68}$Zn(*p*,2*p*)$^{67}$Cu	$^{68}$Zn(*p*,2*n*)$^{67}$Ga	$^{68}$Zn(*p*,2*n*)$^{67}$Ga	$^{68}$Zn(*p*,2*n*)$^{67}$Ga	$^{68}$Zn(*p*,3*n*)$^{66}$Ga	$^{68}\hbox {Zn}(p,x)^{65}\hbox {Zn}$
[MeV]	[mb]	[mb]	[mb]	[mb]	[mb]	[mb]	[mb]
	Method I	Method II	Method I	Method II	Unseparated		
100.09 ± 0.05	12.21 ± 0.55	13.11 ± 0.56	28.24 ± 1.26	25.17 ± 1.07	31.24 ± 1.44	12.43 ± 0.66	98.03 ± 7.92
94.99 ± 0.09	11.76 ± 0.51	12.83 ± 0.53	29.25 ± 1.27	25.91 ± 1.06	30.97 ± 1.40	12.76 ± 0.66	100.01 ± 7.73
89.64 ± 0.4	12.00 ± 0.52	13.06±0.54	31.40±1.36	27.71±1.13	32.95±1.49	13.78 ± 0.71	109.51 ± 8.46
84.06 ± 0.52	10.26 ± 0.44	11.31 ± 0.47	29.40 ± 1.27	26.85 ± 1.09	33.54 ± 1.52	13.86 ± 0.73	112.45 ± 8.54
79.95 ± 0.65	11.12 ± 0.48	12.58 ± 0.51	35.78 ± 1.55	31.13 ± 1.26	39.34 ± 1.79	16.04 ± 0.84	134.62 ± 9.65
75.71 ± 0.66	9.87 ± 0.44	10.70 ± 0.46	35.03 ± 1.61	31.86 ± 1.37	37.40 ± 1.78	17.25 ± 0.91	134.17 ± 0.17
71.30 ± 0.75	9.54 ± 0.45	10.63 ± 0.46	36.34 ± 1.66	33.25 ± 1.42	40.89 ± 1.94	18.66 ± 0.98	148.85 ± 10.47
66.64 ± 0.82	9.46 ± 0.51	10.97 ± 0.58	39.60 ± 2.18	35.36 ± 1.84	42.44 ± 2.39	19.78 ± 1.23	159.87 ± 12.28
61.71 ± 1.05	8.95 ± 0.47	10.65 ± 0.55	45.39 ± 2.50	40.18 ± 2.08	50.70 ± 2.84	24.53 ± 1.48	193.25 ± 14.99
56.47 ± 1.00	7.20 ± 0.55	5.46 ± 0.42	44.24 ± 3.49	46.66 ± 2.08	47.86 ± 3.76	29.09 ± 2.36	231.71 ± 20.85
50.86 ± 1.38	8.26 ± 0.63	10.10 ± 0.77	67.94 ± 5.37	60.99 ± 4.64	71.18 ± 5.60	52.29 ± 4.17	278.29 ± 25.06
47.31 ± 1.25	7.24 ± 0.52	9.85 ± 0.70	74.49 ± 5.53	65.80 ± 4.66	83.93 ± 6.25	77.54 ± 5.81	221.32 ± 21.02
43.54 ± 1.27	6.98 ± 1.59	9.48 ± 2.16	89.76 ± 20.52	82.91 ± 18.85	94.01 ± 21.51	111.07 ± 25.41	120.99 ± 29.68
39.67 ± 1.79	5.81 ± 1.32	10.31 ± 2.34	120.05 ± 27.38	109.25 ± 24.77	1118.61 ± 27.11	118.75 ± 27.14	35.93 ± 10.82
35.36 ± 1.62	2.99 ± 0.81	9.15 ± 2.46	145.71 ± 39.39	130.44 ± 35.09	146.02 ± 39.44	54.98 ± 14.85	−

### $^{68}$Zn(*p*,2*n*)$^{67}$Ga reaction

**Figure 2:**
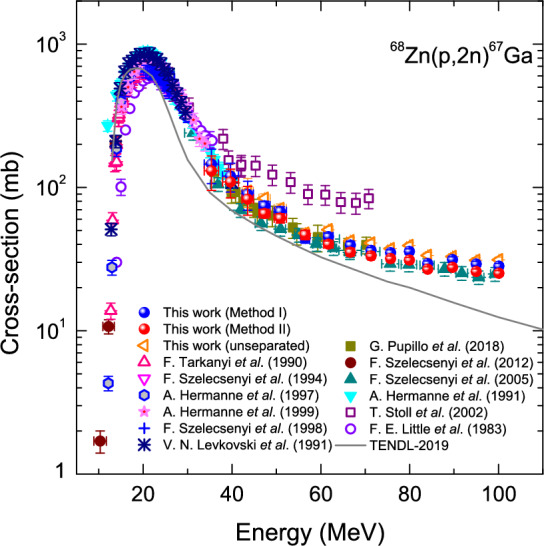
Measured excitation functions of the $^{68}$Zn(*p*,2*p*)$^{67}$Ga reaction as a function of incident proton energy. We plot the data using the developed separation processes for the superposed $\gamma$-ray as denoted by Method I and Method II. Besides, the excitation function obtained without the peak separation is shown for comparison. We include theoretical calculations using a statistical model codes as denoted by TENDL-2019 as well as the data from literature.

As far as the $^{68}$Zn(*p*,2*n*)$^{67}$Ga reaction is concerned, the available data from the literature are more than those of the $^{68}$Zn(*p*,2*p*)$^{67}$Cu reaction, as shown in Fig. 2^4,5,18–21^. In most previous works, they neglect the contribution of $^{68}$Zn(*p*,2*p*)$^{67}$Cu in obtaining the cross-section of $^{68}$Zn(*p*,2*n*)$^{67}$Ga due to its relatively smaller cross sections, in which they acquired the data using the radiochemical separation, unlike in the present work. In the energy range of 40 MeV up to 100 MeV, the present data obtained by Method II is in good agreement with that provided by Szelecsényi et al.^21^ within their quoted uncertainties. For a partial energy range of $\sim$40 MeV up to 70 MeV, the present data by Method I is in good agreement with that of Pupillo et al.^5^ within the uncertainties quoted by the latter. On the other hand, the measurements provided by Stoll et al.^4^ are consistently greater than those of the present over the entire energy range.

Since Szelecsényi et al.^21^ did not perform the chemical separation, their results contain the contribution of the $^{68}$Zn(*p*,2*p*)$^{67}$Cu reaction, i.e., the values do not arise from pure (*p*,2*n*) cross sections. That is why we cannot find their data systematically measured above $\sim$70 MeV in Fig. 1. In later years, Szelecsényi et al.^3^ measured the reaction below 40 MeV after being subjected to the radiochemical separation (see Fig. 1). Comparing the present data of $^{68}$Zn(*p*,2*n*)$^{67}$Ga reaction with the literature, the data obtained from Method I or Method II are in more agreement with that underwent the radiochemical separation^5^ than that did not^21^.

On the other hand, the present data of the unseparated in Fig. 2 arise from the peak at $E_{\gamma }$ = 300.2 keV ($I_{\gamma }$ = 16.64% for $^{67}$Ga) (see Table 2), as in the data obtained from the same peak without the radiochemical separation from that of Szelecsényi et al^21^. Note that the peak at 300.2 keV for $^{67}$Ga exhibits much greater intensity than that of $^{67}$Cu ($I_{\gamma }$ = 0.797%), so the peak was chosen for comparison, unlike the peak separation analyses being used for the peaks at 184.6 keV and 93.3 keV. Obviously, the present data is slightly greater than those of Szelecsényi et al. but is in good agreement with that of Pupillo et al.^5^ up to $\sim$70 MeV.

### $^{68}$Zn(*p*,*x*)$^{65}$Zn reaction

**Figure 3:**
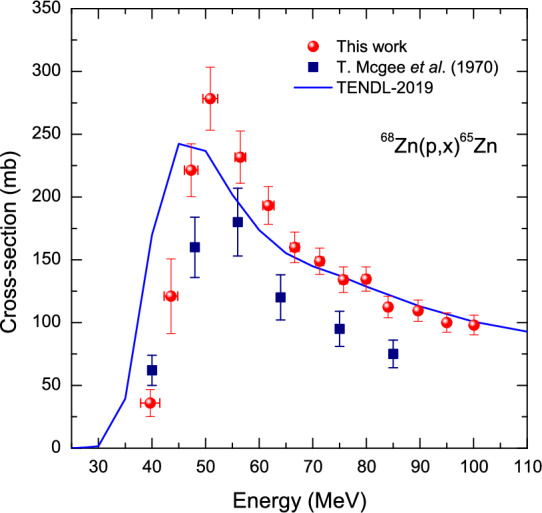
Measured excitation function of the $^{68}$Zn(*p*,*x*)$^{65}$Zn reactions as a function of incident proton energy. We include theoretical calculations using a statistical model codes as denoted by TENDL-2019 as well as the data from literature.

The reaction $^{68}$Zn(*p*, 4*n*)$^{65}$Ga may lead to $^{65}$Zn via the decay of the 15 min $^{65}$Ga. In addition, it is possible to produce $^{65}$Zn by $^{68}$Zn(*p*, *p*3*n*) reaction. Thus, the cross-section of $^{68}$Zn(*p*,*x*)$^{65}$Zn is the sum of the (*p*, 4*n*) and (*p*, *p*3*n*) reactions. In Fig. 3, we may compare the present data with that only from McGee et al.^22^ who obtained the data by radiochemical separation. Above $\sim$47 MeV, the measurements provided by McGee et al. are consistently greater than those in the present work. On the other hand, below $\sim$47 MeV, the data is in good agreement with that of them within their quoted uncertainties. As far as the TENDL-2019 is concerned, above $\sim$66 MeV the present data is in good agreement with the simulated data within our estimated uncertainties.

### $^{68}$Zn(*p*,3*n*)$^{66}$Ga reaction

**Figure 4:**
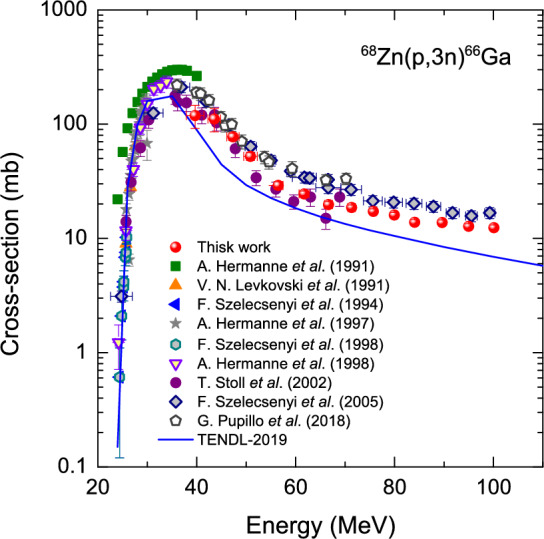
Measured excitation function of the $^{68}$Zn(*p*,3*n*)$^{66}$Ga reactions as a function of incident proton energy. We include theoretical calculations using a statistical model codes as denoted by TENDL-2019 as well as the data from literature.

As far as the $^{68}$Zn(*p*,3*n*)$^{66}$Ga reaction is concerned, we may compare the present data with those obtained by only McGee et al.^22^ and Szelecsényi et al.^21^ that the works systematically present the data up to 100 MeV. In comparison with the data given by Szelecsényi et al.^21^, the present data show slightly lower values above $\sim$47 MeV, as shown in Fig. 4. On the other hand, in the range of 36 MeV to 71 MeV, the present data is in good agreement with that of Stoll et al. within their quoted uncertainties^4^. They estimated the beam flux via the ^nat^Cu(*p*,*xn*)$^{62,63}$Zn and $^{27}$Al(*p*,*x*)$^{22}$Na reactions, which are similar to our case of monitoring via the $^{27}$Al(*p*,*x*)$^{24}$Na reaction. In a narrow energy range of 36 MeV to 47 MeV, the present data is in good agreement with the literature only except for the data from Hermanne et al.^23^.

## Conclusions

In summary, the superposed $\gamma$-ray peaks on $^{67}$Cu and $^{67}$Ga make it difficult to measure the respective $^{68}$Zn(*p*,2*p*)$^{67}$Cu and $^{68}$Zn(*p*,2*n*)$^{67}$Ga reactions. Thus, most previous works exploit a radiochemical separation analysis, which is always concomitant with a loss in separation efficiency. Unlike in previous works, we have developed a new peak separation analysis for the $^{68}$Zn(*p*,2*p*)$^{67}$Cu and $^{68}$Zn(*p*,2*n*)$^{67}$Ga reactions in this work. This newly developed method may allow to leave out the radiochemical separation process, and thus a very efficient way to obtain the reactions, which are universally applied to any superposed $\gamma$-ray spectra. For the first time, two nuclear data of the $\gamma$-ray intensity and the half-life for each nuclide are employed to develop the analysis, by which we measured the cross sections and compared them with those of the previous works. In addition, we employed another separation method which was developed in our previous works and then compared it with other data. Cross sections obtained with the two analytical methods are in good agreement with some of the previous works. For $^{68}$Zn(*p*,2*n*)$^{67}$Ga reaction, we further measured the data without the separation, as in that of previous works, which makes sense due to the value of the reaction being greater than that of $^{68}$Zn(*p*,2*p*)$^{67}$Cu reaction. The cross-section reactions of $^{68}$Zn(*p*,*x*)$^{65}$Zn and $^{68}$Zn(*p*,3*n*)$^{66}$Ga were also measured in the present work, and are in good agreement with some previous works.

## Methods

### Materials and sample preparation

**Figure 5:**
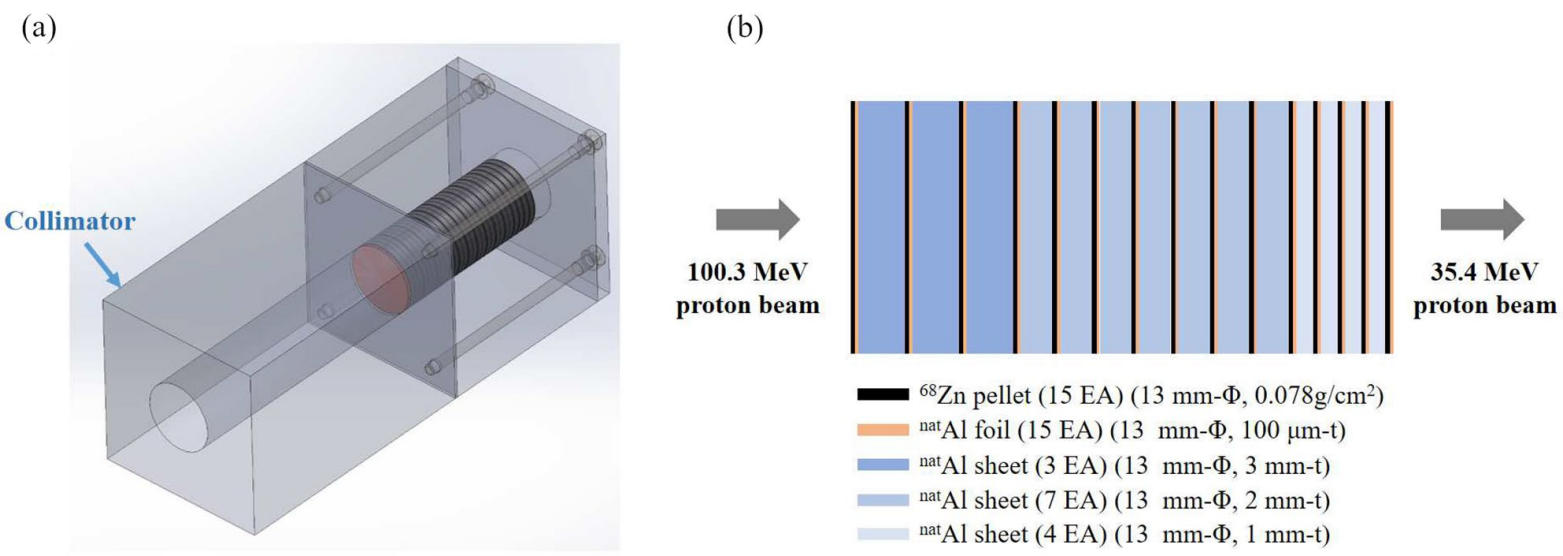
(**a**) Schematic configuration of the stacking foils inside the sample structure. (**b**) Detailed arrangement of the samples stacked in the order of Al foils and Zn pellets, followed by the energy degraders, i.e., thick Al foils. A collimator with a 10 mm diameter hole was followed by the stacked samples, whose diameter were 13 mm. The thickness of the foils in the illustration were depicted to be greater that their actual thickness for clarity.

High-purity-natural aluminum foils were purchased from Goodfellow (Huntingdon, UK), and used for proton beam monitors and energy degraders. Fifteen ^nat^Al foils (99.9% purity) with a thickness of 101.3 ± 0.4 μm and a diameter of 12.91 ± 0.01 mm were used for the proton beam monitor. For energy degradation, ^nat^Al sheet (99.0% purity) with a thickness of 977.6± 5.5 μm and a diameter of 12.84 ± 0.04 mm were used. Enriched $^{68}$Zn metal powder for measuring the cross-section of the radionuclide of interest was purchased from ISOFLEX (California, USA). The foils inside the sample structure consisted of fifteen sets, each set stacking with ^nat^Al foil for beam monitor, pellet disks of enriched $^{68}$Zn, and 2 or 3 sheets of energy degraders, which are behind the collimator ($\Phi$ = 13 mm) (see Fig. 5). We prepared the isotopic composition of the enriched $^{68}$Zn metal powder (99.16%), being compressed into pellets with a diameter of 13.1 mm and a weight of 103.3 ± 1.0 mg. The beam flux was obtained by using the reference reaction of ^nat^Al(*p*,*x*)$^{24}$Na recommended by the International Atomic Energy Agency (IAEA). In Table 4, we also summarize the threshold energies and Q-values for these four kinds of nuclear reactions such as $^{68}$Zn(*p*,2*p*)$^{67}$Cu, $^{68}$Zn(*p*,2*n*)$^{67}$Ga, $^{68}$Zn(*p*,*x*)$^{65}$Zn, and $^{68}$Zn(*p*,3*n*)$^{66}$Ga^24^.

**Table 4:** Threshold energies to produce the $^{67}$Cu, $^{67}$Ga, $^{66}$Ga, and $^{65}$Zn calculated from Q-value calculator^24^.

	Reaction channel on $^{68}$Zn target	Q-value (MeV)	Threshold (MeV)
$^{67}$Cu	*p*,2*p*	$-$ 9.9765	10.1245
$^{67}$Ga	*p*,2*n*	$-$ 11.9847	12.1594
$^{65}$Zn	*p*,*p*+3*n*	$-$ 28.3090	28.7289
	*p*,*d*+2*n*	$-$ 26.0845	26.4714
	*p*,*t*+*n*	$-$ 19.8272	20.1213
$^{66}$Ga	*p*,3*n*	$-$ 23.2084	23.5527

### Proton beam irradiation

Proton beam irradiation was performed at the Korea Multipurpose Accelerator Complex (KOMAC) facility using a 35-100 MeV proton beam to the sample structure consisting of stacked foils and pellets with the collimator as mentioned above. During irradiation, the linear accelerator was operated for $\sim$20 min with a repetition rate of 1 Hz, and an average beam current of $\sim$100 nA. The incident proton beam energy was measured by a multi-layer faraday cup (MLFC, Pyramid MLFC-128-125)^25^ and the beam energy irradiated on each foil and each pellet disk was calculated using the code SRIM-2013^26^.

### Gamma-ray spectroscopy

**Figure 6:**
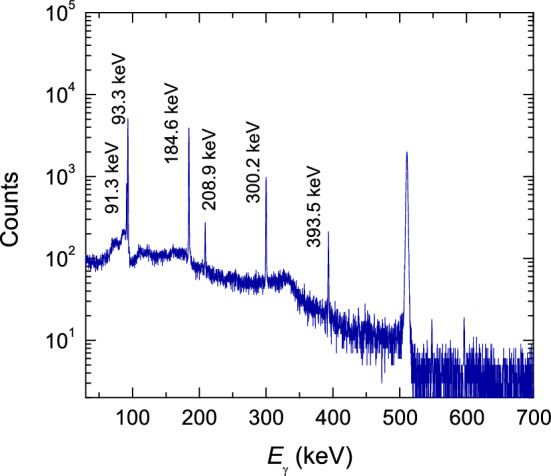
Typical $\gamma$-ray spectrum for the activated $^{68}$Zn pellets, in which the respective $\gamma$ transition peaks are labeled. The spectrum was obtained under the following conditions: $E_{p}$= 100.09 MeV, $t_{i}$= 23.3 min, $t_{w}$= 69.58 h, and $t_{m}$= 10 min.

The $\gamma$-ray spectra for the samples were obtained after a sufficient cooling time of 40 h, which were measured once again passing after as much as a half-life of the radionuclides. All samples were measured with the same *p*-type coaxial high-purity germanium (HPGe) detector coupled with a PC-based 8 k channel analyzer. The energy resolution and relative efficiency of the HPGe detector were 0.875 keV in full-width at half-maximum linewidth and 30% at 122 keV, respectively. To prevent Compton scattering and pile-up effects, the samples were placed as far as 25 cm from the detector surface. We kept the dead time to be less than 5% for all samples. For energy and efficiency calibration of the HPGe detector, they were used that several certified reference materials such as $^{241}$Am, $^{152}$Eu, and $^{137}$Cs. The Genie-2000 software was used to analyze $\gamma$-ray spectra. A typical $\gamma$-ray spectrum of an activated $^{68}$Zn pellet is displayed in Fig. 6.

### Uncertainty

The total uncertainty in the present work was estimated by the square root of the quadratic sum of both statistical and systematic errors^6^. The statistical error in the observed activity is coming from the $\gamma$-ray counting, which was found to be 0.34–4.27%. The systematic errors are due to uncertainties in the detection efficiency (1.54–4.58%), nuclear spectroscopic data (0.02–1.24%), sample thickness (1.08%), and the beam flux (3.96–26.92%), where the error of the standard cross section was not included in our results. Thus, the overall uncertainty of the measured cross sections was estimated to be 4.33–30.12%, including the contribution of the parent nuclide. The energy uncertainty of the incident beam for each foil was estimated to be 0.05–4.58% from the results of the SRIM code.

## Data Availability

The datasets used and/or analyzed during the current study are available from the corresponding author on reasonable request.
